# Case Report: Pathological diagnosis of left upper lobe nodules: two cases of composite small cell lung cancer and literature review

**DOI:** 10.3389/fonc.2025.1629307

**Published:** 2025-10-08

**Authors:** Jishen Zhang, Defeng Jin, Yutao Wei

**Affiliations:** ^1^ School of Clinical Medicine(Affiliated Hospital), Jining Medical University, Jining, China; ^2^ Department of Thoracic Surgery, Jining No. 1 People’s Hospital, Jining, China; ^3^ Institute of Thoracic Diseases, Jining Medical Sciences Academy, Jining, China

**Keywords:** combined small cell lung cancer, small cell lung cancer, pathology, lung cancer, immunotherapy, EGFR mutations, case report

## Abstract

Combined Small Cell Lung Cancer (C-SCLC) is defined by the World Health Organization (WHO) as a combination of Small Cell Lung Cancer (SCLC) and other components, which may include any type of Non-Small Cell Lung Cancer (NSCLC). It is a relatively rare type of lung cancer, with an incidence ranging from 2% to 28%, and its incidence is on the rise. We report two cases of combined small cell lung cancer (C-SCLC) diagnosed by postoperative pathology following thoracoscopic left upper lobectomy at our hospital. One case was small cell carcinoma combined with squamous cell carcinoma (95% SCLC + 5% SCC), while the other was small cell carcinoma combined with large cell carcinoma. Both patients were male with a history of heavy smoking. Postoperatively, they received adjuvant chemotherapy combined with immunotherapy and surgery alone, respectively. No recurrence was observed during follow-up. This paper, in conjunction with a literature review, discusses the clinical and pathological features, diagnosis, potential molecular markers, treatment, and prognosis of C-SCLC. We emphasize the importance of early surgical resection and individualized comprehensive treatment, aiming to provide clinical reference for the diagnosis and management of this rare type of lung cancer.

## 1 Introduction

According to The 2021 WHO Classification of Lung Tumors, lung cancer is generally divided into two main categories: small cell lung cancer (SCLC) and non-small cell lung cancer (NSCLC). Non-small cell lung cancer includes histological subtypes such as adenocarcinoma, squamous cell carcinoma and large cell carcinoma. Small cell lung cancer is an independent type, typically characterized by its highly malignant features and is considered the most aggressive with the poorest prognosis among lung malignancies (1). Most small cell lung cancers are pure small cell lung cancers(P-SCLC). However, some cases have been found, through histological classification during pathological examination, where small cell carcinoma cells and non-small cell carcinoma cells coexist. According to the World Health Organization (WHO) definition from 2001, this has been classified as Combined Small Cell Lung Cancer(C-SCLC).It is a relatively rare type of lung cancer (2).

Therefore, we report two cases of C-SCLC, where the main reason for admission was “left upper lobe nodule found on routine physical examination.” who underwent video-assisted thoracoscopic surgery (VATS) for left upper lobe resection in our hospital’s thoracic surgery department between July 2024 and December 2024, with pathological diagnosis of combined small cell lung cancer (C-SCLC).

## 2 Case report

### 2.1 Case 1

**Figure 1:**
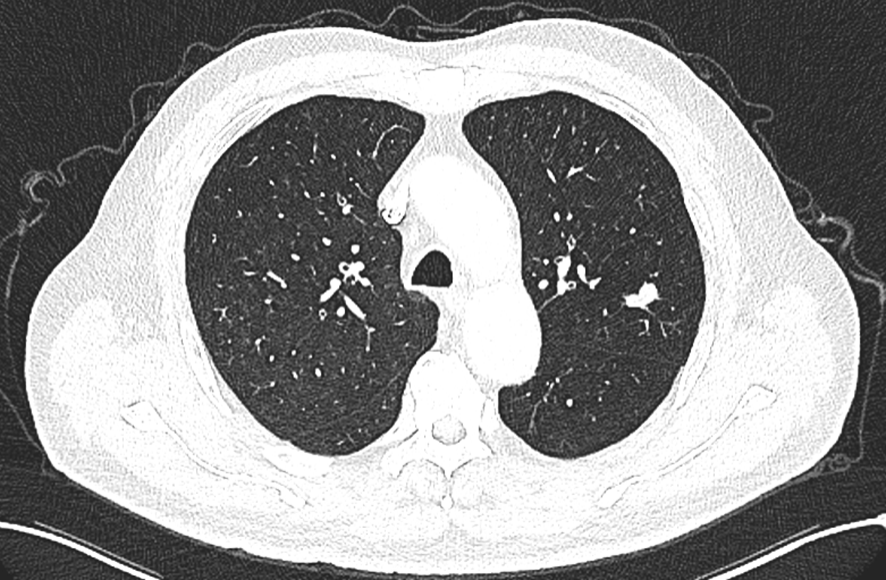
A nodule in the left upper lobe, with the edges appearing lobulated.

**Figure 2:**
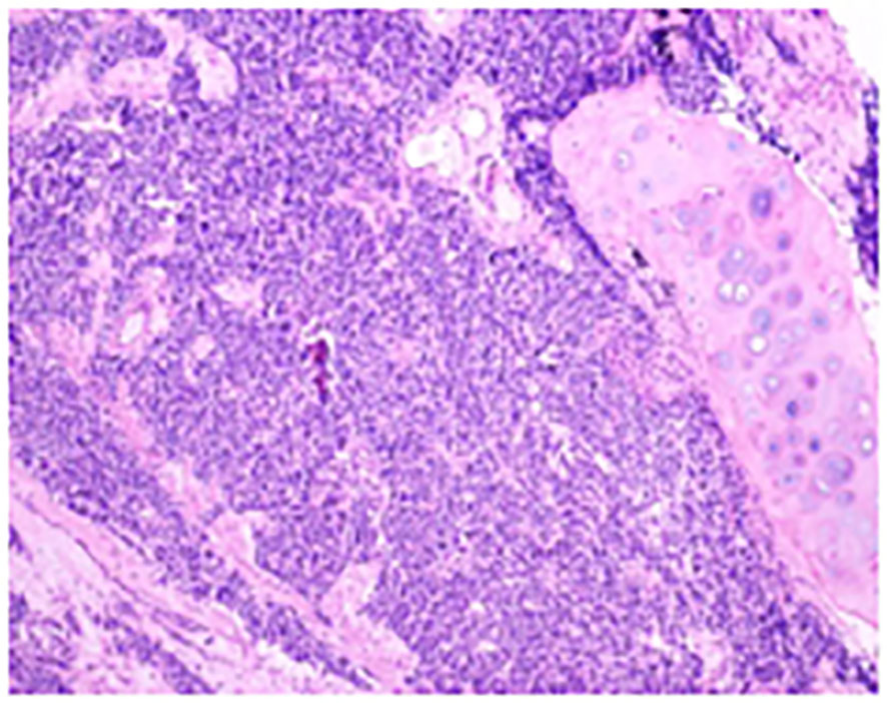
The conventional paraffin pathology of the left upper lobe nodule indicates the coexistence of small cell lung cancer (SCLC) and squamous cell carcinoma (SCC) components in the pulmonary tissue.

A 66-year-old male patient was admitted to our hospital’s thoracic surgery department on November 5, 2024. Due to a “left lung upper lobe nodule discovered during a routine physical examination for over one month.” The patient had a chest CT scan at a physical examination center one month ago, which revealed a nodule in the posterior segment of left upper lobe. A follow-up enhanced chest CT at our hospital showed multiple nodules in both lungs, with left upper lobe being the most prominent, measuring approximately 1.2 cm × 0.6 cm, and the margin appearing lobulated. Other small nodules with a diameter of less than 0.5 cm, recommended for follow-up observation (**Figure 1**). The patient reported having a morning cough with white, sticky sputum. He has a history of cerebral infarction for 8 years and hypertension for over 10 years. The patient also has a long history of heavy smoking and drinking for over 40 years. Physical examination upon admission showed no significant abnormalities. Liver and kidney function tests indicated: total protein 59.1 g/L, albumin 36.5 g/L, with no other abnormalities. Routine blood, urine, stool, thyroid function, coagulation, Arterial blood gas analysis and cardiac enzyme tests were normal. Pulmonary function tests showed forced expiratory volume in one second/forced vital capacity (FEV1/FVC) of 69.5%, FEV1 of 1.76 L (63.4% of predicted value), total lung capacity (TLC) at 99.5% of predicted value, and diffusing capacity of the lungs for carbon monoxide (DLCO) at 93.4% of predicted value, indicating moderate obstructive ventilatory dysfunction. Electrocardiogram showed: 1. sinus rhythm, 2. abnormal Q waves (II, III, aVF), 3. incomplete right bundle branch block. Coronary CTA showed severe stenosis in the mid-segment of left anterior descending artery and diagonal branch, with the remaining coronary arteries showing mild stenosis. Brain MRI showed mild brain atrophy. Ultrasound examinations of the heart, liver, gallbladder, pancreas, spleen, kidneys, neck arteries, and lower extremity veins, along with whole-body bone scintigraphy, showed no significant abnormalities. After excluding contraindications for surgery, the patient underwent thoracoscopic left upper lobectomy under general anesthesia on November 11. Due to the diameter of other small lung nodules being less than 0.5 cm, regular follow-up observation is recommended and no intervention was performed during the surgery. Intraoperative dissection of lymph nodes from groups 5, 7, 10 and 11. Intraoperative frozen section pathology indicates invasive carcinoma and subsequent routine paraffin-embedded pathology (**Figure 2**) showed immunohistochemistry results: CK5/6 (partially +), P40 (partially +), P63 (partially +), CK7 (focally +), Napsin A (-), TTF-1 (+), Ki67 (+, approximately 70%), CD56 (+), CgA (-), Syn (+). Based on immunohistochemistry, the diagnosis was composite small cell carcinoma (95% small cell carcinoma + 5% squamous cell carcinoma). The tumor measured approximately 1.4 × 1 × 0.7 cm and had not invaded pleura. No metastatic cancer was found in lymph nodes. According to pathological report, the staging was T1bN0M0 IA2. After surgery, the patient received one cycle of chemotherapy and immunotherapy with the following regimen: albumin-bound paclitaxel 300 mg, carboplatin 700 mg, and serplulimab 300 mg. After discharge, the patient reported no significant discomfort. Chest CT re-examination at 1^st^ and 3^rd^ month post-surgery showed no significant abnormalities (**Table 1**).

**Table 1:** Clinical features, pathological types, treatment methods, and follow-up of two C-SCLC patients.

Basic Information of Patients	Case1	Case2
Gender	Male	Male
Age	66 years	58 years
History of Heavy Smoking	Yes	Yes
Symptoms	Morning cough, expectorating white mucus	None
Location of Lung Nodules	Left upper lobe	Left upper lobe
Size of Lung Nodules	1.2cm×0.6cm	2.1cm×1.6cm
Pathological Type	Composite small cell carcinoma (95% small cell carcinoma + 5% squamous cell carcinoma)	Composite small cell carcinoma (neuroendocrine carcinoma + large cell carcinoma)
Lymph Node Metastasis	No metastatic cancer observed	Metastatic cancer found in lymph nodes of group 6 and group 11, no metastasis in other groups
Treatment Method	Left upper lobe resection + 1 cycle of chemotherapy combined with immunotherapy; regimen: albumin-bound paclitaxel 300mg, carboplatin 700mg, surufatinib 300mg	Left upper lobe resection
Follow-up	Chest CT re-examination at 1st and 3rd month post-surgery showed no significant abnormalities	Chest CT re-examination at 1st and 10th month post-surgery showed no significant abnormalities

### 2.2 Case 2

**Figure 3:**
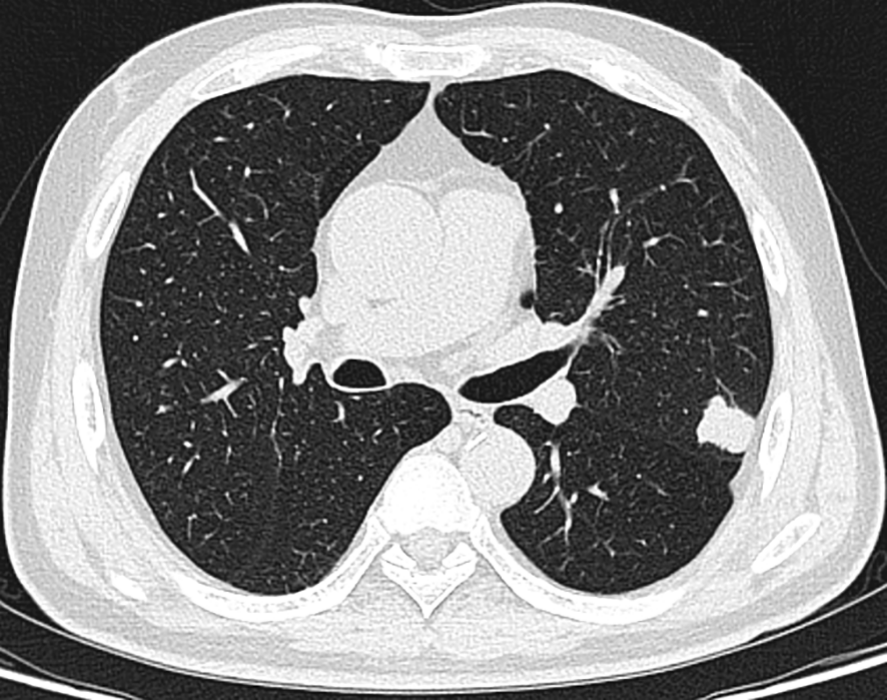
Lobulated nodule in the left upper lobe.

**Figure 4:**
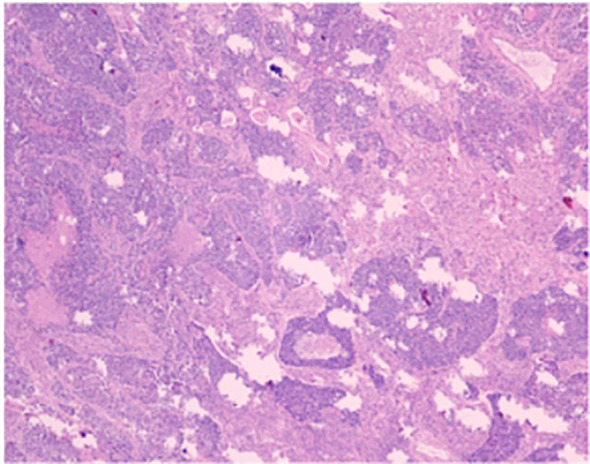
The conventional paraffin pathology of the left upper lobe nodule indicates the coexistence of small cell lung cancer (SCLC) and large cell lung cancer (LC).

The patient is a 58-year-old male who was admitted to our hospital’s thoracic surgery department on July 26, 2024, due to a “6-month history of a left lung upper lobe nodule discovered during a routine examination.” Approximately 6 months ago, during a routine physical examination at another hospital, the patient underwent a chest CT scan which revealed multiple lung nodules, the largest of which was located in left upper lobe. He subsequently underwent regular follow-up scans. A chest CT scan performed at our hospital showed a lobulated small nodule in left upper lobe, measuring approximately 2.1 cm × 1.6 cm. Multiple small nodules in both lungs, with the largest measuring approximately 0.5 cm, recommended for follow-up examination. No significant enlargement of lymph nodes in mediastinum was observed (**Figure 3**). The patient reported no symptoms. He has a 10-year history of hypertension and a 30-year history of heavy smoking and drinking. There were no significant findings during physical examination upon admission. Blood gas analysis showed: pH 7.48, blood glucose 10.70 mmol/L, K^+^ 2.80 mmol/L. Routine blood, urine, and stool tests, liver and kidney function, and coagulation profile were normal. Pulmonary function testing showed FEV1 of 2.76L, indicating mild obstructive ventilatory dysfunction. The electrocardiogram showed no abnormalities. Coronary CTA revealed moderate stenosis in the proximal left anterior descending artery, mild stenosis in the proximal left circumflex artery, and mild stenosis in the right coronary artery in the mid-segment, with no significant narrowing of the other coronary arteries. Brain MRI showed signs of cerebral arteriosclerosis. Ultrasound examinations of the liver, gallbladder, pancreas, spleen, kidneys, adrenal glands, bilateral carotid arteries, bilateral lower extremity veins, and full-body bone scans were all unremarkable. The patient’s family requested surgical treatment for left upper lobe nodule regardless of whether it is benign or malignant. And refused to undergo further relevant tests to clarify the diagnosis. After excluding surgical contraindications, on July 30, the patient underwent a left upper lobectomy under general anesthesia with thoracoscopic surgery. Routine dissection of lymph nodes from groups 5, 6, 7, 9, 10 and 11. Rapid intraoperative frozen section pathology indicated invasive carcinoma and subsequent routine paraffin pathology (**Figure 4**) showed immunohistochemistry: CK5/6 (-), CK7 (partial +), Napsin A (-), P40 (-), P63 (-), TTF-1 (partial +), CD56 (partial +), CgA (-), Syn (partial +), SSTR2 (-), SMARCA4 (+), CK (+), Ki67 (+, approximately 80%). Based on the immunohistochemistry results, the diagnosis was consistent with a composite small cell carcinoma (neuroendocrine carcinoma + large cell carcinoma). The tumor measured approximately 2.5 × 2 × 2 cm and did not invade pleura. Metastatic cancer was found in the 6^th^ group lymph nodes (2/2) and the 11^th^ group lymph nodes (1/7), no metastatic cancer was observed in other lymph node groups. Based on pathology report, the clinical stage was T1cN2bM0, which corresponds to stage IIIA. Postoperatively, no special treatment was given. After discharge, the patient reported no significant discomfort. Chest CT re-examination at 1^st^ and 10^th^ month post-surgery showed no significant abnormalities (**Table 1**).

## 3 Discussion

The combined small cell lung cancer (C-SCLC) reported by us is defined by the World Health Organization (WHO) as a combination of small cell carcinoma (SCLC) and other components, which include any type of non-small cell lung cancer (NSCLC). It is a relatively rare type of lung cancer (2, 3).The incidence of combined small cell lung cancer reported in different studies ranges from 2% to 28%. With the increasing role of surgical resection in the treatment of early to mid-stage lung cancer, the incidence of combined small cell lung cancer has shown an upward trend (4).

Most patients with combined small cell lung cancer (C-SCLC) are male, with a proportion ranging from 43% to 82.5%. This phenomenon is likely closely related to smoking, as nearly all C-SCLC patients have a history of heavy smoking (5). The two cases of C-SCLC that we reported were both male patients with a history of heavy smoking. Previously, Nicholson et al. reported that among 43 cases of combined small cell lung cancer (small cell lung cancer combined with squamous cell carcinoma, SCLC/SCC), only 2 cases were non-smokers, with the remaining 41 having a history of heavy smoking (6). Furthermore, Luo et al. reported that among 80 cases of combined small cell lung cancer (SCLC/SCC), 62 patients had a history of smoking, accounting for 77.5% (7). In a study by Lu HY et al. that 71.4% of the 7 C-SCLC patients were male, and 71.4% were heavy smokers (8). The main clinical symptoms of combined small cell cancer patients are cough, dyspnea, and hemoptysis, often accompanied by malignant pleural effusion. The common location is primarily in upper lobe of the lung and it is located in central region of the lung. But some cases also occur in peripheral region of the lung. Previously, Moon SW et al. reported that composite small cell lung cancer was mainly located in upper lobe of the lung (9). In our report, the lesions in both cases of C-SCLC were located in peripheral region of left upper lobe. Men Y et al. reported that in 114 cases of C-SCLC, tumors in 92 patients were located in central region (10). Luo et al.’s study found that in 88 cases of C-SCLC, 76 patients had imaging findings of central-type mass lesions (11). However, Mangum et al. reported that patients with histological components of C-SCLC had a higher incidence of peripheral lesions on chest X-rays, accounting for 56% (12). In summary, the majority of C-SCLC patients have tumors located in the central region on imaging. But if a patient has a history of heavy smoking, pleural effusion with small cell carcinoma components, and peripheral lesions on imaging, we should consider the possibility of C-SCLC.

Combined small cell lung cancer (C-SCLC) typically exhibits two main pathological components, usually including typical small cell lung cancer cells and various subtypes of non-small cell lung cancer. In the two cases of C-SCLC that we reported, one had a pathological composition of 95% small cell carcinoma and 5% squamous cell carcinoma, while the other had small cell carcinoma and large cell carcinoma. Previously, Babakoohi S et al. reported that among 428 small cell lung cancer patients, 22 were diagnosed with combined small cell lung cancer. Of these, 16 cases had a pathological type of small cell carcinoma combined with large cell carcinoma (SCLC/LC), which was the most common pathological type. This was followed by small cell carcinoma combined with squamous cell carcinoma (SCLC/SCC) in 2 cases, small cell carcinoma combined with adenocarcinoma (SCLC/ADC) in 1 case, and 2 cases where the non-small cell carcinoma component was not clearly identified, classified as small cell carcinoma with an undefined type of non-small cell lung cancer (13). Lu HY et al. reported 7 cases of C-SCLC, of which 5 were small cell carcinoma combined with adenocarcinoma (SCLC/ADC), and 2 were small cell carcinoma combined with squamous cell carcinoma (SCLC/SCC) (8). Men Y et al. reported 114 cases of C-SCLC, with the most common combined component being squamous cell carcinoma (SCC) in 60 cases (52.6%), followed by adenocarcinoma (ADC) in 37 cases (32.5%) and large cell carcinoma (LCC) in 13 cases (11.4%) (10). In summary, the most commonly associated non-small cell lung cancer subtypes in combined small cell lung cancer are squamous cell carcinoma (SCC), adenocarcinoma (ADC), and large cell carcinoma (LCC).

Currently, there are no established standardized molecular markers for combined small cell lung cancer (C-SCLC). Epidermal Growth Factor Receptor (EGFR) is a cell surface receptor protein belonging to the tyrosine kinase receptor family. It participates in fundamental processes such as cell proliferation, differentiation, and survival. Upon activation in normal cells, it phosphorylates its intracellular domain, which in turn activates the downstream PI3K/Akt and MAPK/ERK pathways, promoting the proliferation, migration, and invasion of tumor cells (14). EGFR is typically important in various cancers, particularly in non-small cell lung cancer (NSCLC). Mutations in the EGFR gene lead to excessive or sustained activation of the receptor, which becomes one of the driving forces for the malignant proliferation of cancer cells. The vast majority of EGFR mutations occur in lung adenocarcinoma, with some mutations also found in other subtypes, such as adenosquamous carcinoma. However, such mutations are rare in small cell lung cancer (SCLC) (15). Nevertheless, some studies have suggested that EGFR mutations may also exist in C-SCLC patients. Lu HY et al. used xTAG technology to evaluate EGFR mutations in 40 SCLC patients and found 2 cases of EGFR mutations located in exon 19. One case was a non-smoker, with a pathological type of small cell carcinoma combined with adenocarcinoma (SCLC/ADC) in a female; the other case was a smoker, with a pathological type of small cell carcinoma combined with squamous cell carcinoma (SCLC/SCC) in a male (8, 16). Previously, Li et al. performed genetic testing on 98 surgical samples, among which 11 patients (11.2%) exhibited EGFR mutations, all of which were observed in C-SCLC (17). Tatematsu A et al. assessed the EGFR mutation status in 122 SCLC patients, including 15 C-SCLC patients, and identified EGFR mutations in 3 C-SCLC (SCLC/ADC) patients with a light smoking history, where both the SCLC and ADC components had EGFR mutations (15). Therefore, whether EGFR can be considered a potential molecular marker for C-SCLC requires further investigation. Currently, targeted therapies for EGFR gene mutations are still relatively limited. Previously, Fu K et al. reported that osimertinib was recommended as the standard first-line treatment for advanced or metastatic NSCLC patients with EGFR mutations, although acquired resistance is inevitably developed (18). Billowria K et al. demonstrated, through animal model experiments, that a novel bispecific antibody, Amivantamab, shows efficacy in treating EGFR Exon 20 insertion mutations in non-small cell lung cancer (19). However, these are preclinical data, and the effectiveness in clinical practice for EGFR-mutant patients requires further investigation. Pal R et al. recently reported that 4-(4-ethoxyphenyl)-6-(substituted-phenyl)pyrimidin-2-amine/thiol/hydroxy series compounds RT-8 and RT-11 exhibit strong inhibitory activity against both EGFRWT and EGFRT790M, with favorable binding modes, laying a foundation for future EGFR inhibitor research. However, their efficacy as novel candidates for EGFRWT and EGFRT790M remains to be further studied (20). Given that C-SCLC contains NSCLC components, the application of treatment regimens for EGFR-mutant NSCLC patients to C-SCLC patients with EGFR mutations still requires further exploration.

C-SCLC is a rare type of lung cancer and its clinical presentation and treatment response may be influenced by tumor composition. As a result, the treatment plan for C-SCLC has not been fully established. The currently common treatment strategies involve chemotherapy, immunotherapy, radiotherapy, and surgery, as part of a multidisciplinary approach. Multiple studies have confirmed that surgery plays a crucial role in improving the treatment outcomes for C-SCLC patients and is the most commonly chosen therapeutic approach by clinicians in clinical practice (10, 13, 21, 22). In the two cases of C-SCLC that we reported, after completing relevant examinations and excluding surgical contraindications, both patients underwent surgical resection of left upper lobe. The surgeries were successful and both patients recovered and were discharged. Babakoohi S et al. compared 22 cases of C-SCLC with 406 cases of pure SCLC and found that the surgery treatment rate in the C-SCLC group was higher than in the pure SCLC group (45% vs. 3%; *P* < 0.0001), and the overall survival (OS) of C-SCLC patients was significantly higher than that of pure small cell lung cancer (SCLC) patients (13). Men Y et al. reported a 5-year OS of 48.9% in C-SCLC patients who underwent surgery, which was significantly higher than the 36.6% in the non-surgical group (10). Lei et al. reported that the median DFS and OS for 181 patients with stage I-IIIa C-SCLC who underwent radical surgery were 32.5 and 49.7 months, respectively. The 1-year, 3-year, and 5-year disease-free survival rates for entire cohort were 68.5%, 32.6%, and 16.0%, respectively (23). He et al. retrieved data from the SEER database for C-SCLC patients and analyzed clinical characteristics, first-line treatment regimens, surgical methods, and survival data, including overall survival (OS) and cancer-specific survival (CSS). They concluded that surgery is beneficial for patients with stage IA-IB C-SCLC. For stage IA-IIIa, the prognosis of C-SCLC is better than SCLC but worse than NSCLC. In stages IIB-IV, there is no difference in prognosis between C-SCLC and SCLC patients regarding surgery (24). Additionally, the surgical resection range of the lesion in C-SCLC patients is also linked to disease prognosis. Guo et al. study demonstrated that lobectomy, compared to sublobar resection, had significantly better prognostic outcomes for C-SCLC patients, with 5-year OS rates of 25% and 68.1%, respectively (*P* < 0.001) (4). Therefore, for early-stage C-SCLC patients, surgery should be performed as early as possible. The resection range should be expanded according to lesion location and systematic lymph node dissection should be carried out to ensure complete tumor removal and reduce the risk of metastasis, thereby extending the patient’s survival period. Regarding chemotherapy’s effect on C-SCLC, Lu HY et al. reported that among seven C-SCLC patients who underwent surgery, one patient who did not receive chemotherapy had a shorter survival compared to other stage IIIA patients who received chemotherapy, indicating the important role of chemotherapy in treating C-SCLC (8). Previously, Rajput, P S et al. reported that the chemotherapeutic agent Lurbinectedin exerts its effects by inhibiting the transcriptional activity of coding genes, thereby suppressing tumor-associated macrophages and impacting the tumor microenvironment. Lurbinectedin has emerged as a potential candidate for treatment of small cell lung cancer (SCLC) (25). However, whether it could serve as a potential candidate for patients with a higher proportion of SCLC components in C-SCLC remains to be further investigated. However, Men Y et al. reported that 84% of C-SCLC patients received chemotherapy, but the 5-year overall survival rate (OS) after chemotherapy was 37.7%, which was similar to the 35.4% in the non-chemotherapy group, with no significant difference between the two groups, suggesting that C-SCLC is relatively less sensitive to chemotherapy drugs (10). In the two cases of C-SCLC that we reported, one patient received one cycle of chemotherapy and immunotherapy one month after surgery, with the regimen consisting of 300 mg albumin-bound paclitaxel, 700 mg carboplatin, and 300 mg serplulimab. The other patient did not receive any special treatment after surgery. Therefore, considering that C-SCLC contains both SCLC and NSCLC components, the importance of chemotherapy in treating C-SCLC and the optimal chemotherapy regimen remains controversial. An individualized treatment plan may need to be developed based on the proportion of SCLC and NSCLC components in C-SCLC.

## 4 Conclusion

In conclusion, these are two cases of C-SCLC that we reported, both of which underwent thoracoscopic left upper lobe resection, with pathological diagnosis of composite small cell lung cancer. Both cases are currently under follow-up and have a good prognosis. The patient received one cycle of chemotherapy and immunotherapy postoperatively and is currently in good condition. This is a case of C-SCLC with a favorable prognosis. At present, for this relatively rare type of lung cancer, clinical characteristics, potential molecular markers and treatment strategies do not yet have clear standardized guidelines. An individualized treatment plan should be developed based on the patient’s specific characteristics.

## Data Availability

The original contributions presented in the study are included in the article/Supplementary Material. Further inquiries can be directed to the corresponding author.
